# The prognostic value of serum albumin levels and respiratory rate for community-acquired pneumonia: A prospective, multi-center study

**DOI:** 10.1371/journal.pone.0248002

**Published:** 2021-03-04

**Authors:** Lili Zhao, Jing Bao, Ying Shang, Ying Zhang, Lu Yin, Yan Yu, Yu Xie, Li Chen, Yali Zheng, Yu Xu, Zhancheng Gao

**Affiliations:** 1 Department of Respiratory and Critical Care Medicine, Peking University People’s Hospital, Beijing, People’s Republic of China; 2 Department of Respiratory and Critical Care Medicine, Xiang’An Hospital of Xiamen University, Xiamen, Fujian, People’s Republic of China; Juntendo University Urayasu Hospital, JAPAN

## Abstract

Community-acquired pneumonia (CAP) is a respiratory disease frequently requiring hospital admission, and a significant cause of death worldwide. This study aimed to investigate the prognostic value of clinical indicators. A prospective, multi-center study was conducted (January 2017–December 2018) where patient demographic and clinical data were recorded (N = 366). The 30-day mortality rate was 5.46%. Cox Regression analyses showed that serum albumin (ALB) and respiratory rate (RR) were independent prognostic variables for 30-day survival in patients with CAP. Albumin negatively correlated with the Pneumonia Severity Index (PSI) and CURB-65 scores using Pearson and Spearman tests. Survival curves showed that a RR >24 breaths/min or ALB ≤30 g/L were associated with a significantly higher risk of mortality. The area-under-the-curve (AUC) for predicting 30-day mortality in patients with CAP was 0.762, 0.763, 0.790, and 0.784 for ALB, RR, PSI, and CURB-65, respectively. The AUC for the prediction of 30-day mortality using ALB combined with PSI, CURB-65 scores, and RR was 0.822 (95% CI 0.731–0.912), 0.847 (95% CI 0.755–0.938), and 0.847 (95% CI 0.738–0.955), respectively. Albumin and RR were found to be reliable prognostic factors for CAP. This combination showed equal predictive value when compared to adding ALB assessment to PSI and CURB-65 scores, which could improve their prognostic accuracy.

## Introduction

Community-acquired pneumonia (CAP) is a common respiratory disease frequently requiring hospital admission, and a significant cause of death worldwide [1–3]. Various papers from many different countries report a wide range of in-hospital mortality values, varying from 4% to 20.9% [3–6]. It is an infectious disease associated with a high mortality rate. Because of the diversity of pathogens and clinical manifestations, the assessment of the severity of disease is necessary for physicians to allocate medical resources early and effectively, and it is important for further therapeutic options and prediction of outcomes [7].

Many CAP-specific score methods for determining disease severity have been developed and used [4,8]. Among them, the Pneumonia Severity Index (PSI) and CURB-65 are the most popular scoring methods used to predict outcomes in patients with CAP [9]. The PSI score method was proposed by Fine and colleagues—it covers more comprehensive factors, but is more cumbersome [10]. The CURB-65 score method was proposed by the British Thoracic Society, and further improved by Lim et al [11,12]. CURB-65 is more concise and convenient to apply to these types of analyses. These scores are useful in the management of patient risk stratification, but there is still a lack of accurate assessment with regard to patient mortality. In addition to the scoring methods, in recent years, selected blood markers have been used to predict outcomes in patients with CAP [13–15]. Traditional indicators such as serum albumin (ALB) [16], C-reactive protein (CRP) [6], lactate [17], glucose level [18], B-type natriuretic peptide [15,19], and troponin were reassessed in a clinical study by Menendez et al. [15]. At the same time, novel biomarkers such as suPAR [20], syndecan-4 [20], soluble triggering receptors expressed on myeloid cells-1 [21], procalcitonin (PCT) [6,22], and pro-adrenomedullin [15,23] have all been validated for determining disease severity in patients with CAP.

Since the patients in different studies came from diverse countries and regions, and patient admission criteria were slightly different depending on the location, a variety of traditional markers were screened for the reassessment of the severity of hospitalized patients with CAP. This study was based on the data from patients with CAP from six different cities in China. It was not only a validation of current CAP-specific score methods, but also aimed to find new possible risk factors or combinations of risk factors to improve the prognostic evaluation of CAP.

## Materials and methods

### Study population

This was an observational analysis of a prospective cohort of patients with CAP hospitalized at Peking University People’s Hospital, West China Hospital, Second Hospital of Jilin University, Shanghai Pulmonary Hospital, Fujian Provincial Hospital, and Tibet Autonomous Region People’s Hospital between January 2017 and December 2018 (ClinicalTrials.gov ID, NCT03093220). The study was approved by the medical ethics committee of Peking University People’s Hospital. All patients provided written informed consent upon enrollment, and were subsequently followed up.

Eligible subjects were above the age of 18 years and CAP was defined as satisfying all the following criteria [24]: (1) symptom onset began in the community; (2) a chest radiograph showed either a new patchy infiltrate, leaf or segment consolidation, ground glass opacity, or interstitial change; (3) at least one of the following signs: (a) the presence of cough, sputum production, and dyspnea; (b) core body temperature >38.0°C; (c) auscultatory findings of abnormal breath sounds and rates; or (d) peripheral white blood cell (WBC) counts >10×10^9^/L or <4×10^9^/L. Patients with tuberculosis, malignant lung tumors, pulmonary interstitial disease, pulmonary embolism, pulmonary vasculitis, human immunodeficiency virus infection, or who were pregnant were excluded.

### Data collection and follow-up

Basic patient information and laboratory data were prospectively collected during their hospital stay by doctors or investigators using a computer-assisted protocol. Each enrolled patient was assigned a unique number. Data were collected on demographic characteristics (e.g., sex, age), clinical symptoms (e.g., cough, difficulty breathing, chest pain), vital signs (e.g., body temperature, respiratory rate, blood pressure, heart rate, consciousness disorder), comorbidities (e.g., chronic pulmonary disease, chronic cardiac disease, chronic renal disease, chronic liver disease, cerebrovascular disease, high blood pressure, diabetes mellitus, malignant diseases), laboratory examinations (routine blood tests, liver function, renal function, electrolytes, blood gas concentration, PCT, CRP, coagulation function within 24 hours after admission), and treatment and prognosis. The loop-mediated isothermal amplification (LAMP) method was used to detect bacteria, viruses, and fungi.

The mortality risk of every patient was evaluated by doctors according to indicators of the PSI and CURB-65 scores. CURB-65 is a five-point scale, where one point is allocated for each symptom of: confusion, urea >7 mmol/L, respiratory rate ≥30 breaths/min, low systolic (<90 mm Hg) or diastolic (≤60 mm Hg) blood pressure, and age ≥65 years. The PSI score was calculated as presented in the study by Fine et al., and comprises the following variables: age, sex, comorbidities, and vital sign abnormalities, together with several laboratory indicators (arterial blood pH <7.35, blood urea nitrogen (BUN) ≥11 mmol/L, blood sodium <130 mmol/L, blood sugar ≥14 mmol/L, hematocrit <30%, and PaO2 <60 mm Hg or oxygen saturation <90%), and radiographic parameters.

All data were inserted into the Peking University People’s Hospital Network Platform of Severe Acute Respiratory Infectious Diseases (http://123.57.13.108/hospital/index.jsp). A total of 401 patients were diagnosed with CAP, of which 35 were excluded due to incomplete data. Eventually, 366 patients with CAP were studied.

### Statistical analysis

Categorical variables, which were described using counts and percentages, were analyzed using either a chi-square, correction for continuity chi-square, or Fisher’s exact test, as appropriate. Normally distributed continuous variables were expressed as means ± standard error from the mean, and non-normally distributed continuous variables were expressed as medians and interquartile ranges. Two groups of continuous equivalent variables with normal distributions were compared using Student’s t-test. Abnormally distributed continuous variables or heterogeneous variables between two groups were compared using the Mann–Whitney U test. Cox proportional hazards regression analyses were used to analyze the effects of an array of variables on 30-day survival. Receiver operating characteristic (ROC) curves were used to evaluate the sensitivity and specificity of different variables on 30-day mortality in patients with CAP. Areas-under-the-curve (AUCs) and optimal threshold values were calculated. Kaplan–‍Meier methods were used to build 30-day survival curves, and survival rates were compared using the log-rank test. The correlation between variables with a normal distribution was assessed using Pearson’s correlation test, while non-normally distributed variables were assessed using Spearman’s rho test.

All statistical analyses were performed using the SPSS v.20.0 software (SPSS Inc., Chicago, IL, USA), MedCalc statistical software v.15.2.2 (MedCalc Software, Ostend, Belgium), and GraphPad Prism version 8 software (GraphPad Software, LaJolla, CA, USA). A two-sided P value <0.05 was considered statistically significant.

The sample size and power calculation for the Cox regression for survival analysis was as follows:

$$N=\frac{({Z_{1-\alpha /2}+Z_{1-\beta })}^{2}}{P(1-R^{2})\sigma^{2}B^{2}}$$

In this study, we set the significance level (two-sided) at α = 0.05. Using the above formula and a sample size of 366, the test standard deviation was Z1-α/2 = 1.96, 30-day mortality P = 0.0546, R-squared of albumin with RR 0.103, standard deviation of albumin σ = 6.16, log hazard ratio of albumin B = -0.098, and calculated power 0.72438.

## Results

### Characteristics of survivors and non-survivors

The clinical characteristics and laboratory findings of the enrolled patients with CAP are presented in Table 1. The 366 patients enrolled from January 2017 to December 2018 were divided into survivor and non-survivor groups. Thirty-day mortality was observed in 5.46% (n = 20) of all patients with CAP. All data were collected within 24 hours of admission, excluding cases with complications and severe CAP. There were no significant differences between the survivors and non-survivors regarding sex, age, comorbidities, mean arterial pressure, body temperature, WBC count, hemoglobin levels, or platelet count. The number of complications (including sepsis, pleural effusion, acute respiratory distress syndrome, and confusion) and cases of severe CAP were significantly higher in the non-survivors than survivors. Initial vital sign analyses showed that the non-survivors exhibited a higher respiratory rate (RR; P<0.001) and heart rate (HR; P = 0.031) than survivors. Laboratory analyses showed that the non-survivor group exhibited higher levels of glucose, BUN, CRP, and PCT than detected in the survivor group. There were no statistical differences in the detection rates of bacteria, viruses, or fungi using the LAMP method. The median ALB in the non-survivor group was 28.6 g/L, which was lower than that in the survivor group (36 g/L; P<0.001). The PSI and CURB-65 scores in the non-survivor group were 97 (85–130) and 2 (1–2), respectively, which were significantly higher than those of patients in the survivor group [75 (57–91) and 0 (0–1), respectively; P<0.001 for the two comparisons].

**Table 1:** Clinical characteristics and laboratory findings of survivors and non-survivors.

	All patients n = 366	Survivors n = 346 (94.54%)	Non-Survivors n = 20 (5.46%)	P value
**Male sex (%)**	227 (62.00%)	213 (61.60%)	14 (70%)	0.450
**Age (years)**	66 (53–76)	65 (52–76)	69 (64–78)	0.070
**Comorbidities, n (%)**				
** Heart disfunction**	24 (6.56%)	23 (6.65%)	1 (5.00%)	1.000
** Chronic renal disease**	14 (3.83%)	14 (4.07%)	0 (0.00%)	1.000
** Liver disease**	20 (5.46%)	20 (5.78%)	0 (0.00%)	0.549
** COPD**	47 (12.84%)	46 (13.29%)	1 (5.00%)	0.463
** Diabetes mellitus**	74 (20.22%)	72 (20.81%)	2 (10.00%)	0.377
** High pressure**	109 (29.78%)	104 (30.06%)	5 (25.00%)	0.631
** Cerebrovascular disease**	15 (4.10%)	14 (4.05%)	1 (5.00%)	0.577
** Malignant disease**	30 (8.20%)	29 (8.38%)	1 (5.00%)	0.907
**Complications**				
** Septicopyemia**	35 (9.56%)	23 (6.65%)	12 (60.00%)	<0.001
** Pleural effusion**	122 (33.33%)	110 (31.79)	12 (60.00%)	0.009
** ARDS**	16 (4.37%)	5 (1.45%)	11 (55.00%)	<0.001
** Confusion**	13 (3.55%)	9 (2.62%)	4 (20.00%)	0.003
**Initial vital signs**				
** Mean arterial pressure (mmHg)**	91 (84–100)	91 (84–98)	91 (78–102)	0.605
** Respiratory rate (/min)**	20 (20–22)	20 (20–22)	26 (20–35)	<0.001
** Heart rate (/min)**	85 (78–96)	84 (78–95)	93(83–106)	0.031
** Body temperature (°C)**	38.2 (36.9–39.0)	38.2 (36.9–39.1)	38.3 (37.0–39.0)	0.966
**Laboratory findings**				
White blood cell count (×10^3^/mm^3^)	6.80 (4.90–10.67)	6.80 (4.90–10.50)	9.45 (4.41–16.76)	0.251
** Hemoglobin level (g/dL)**	129.00 (117.00–141.00)	129.00 (117.00–140.50)	132.50 (114.75–151.75)	0.261
Platelet count (×10^3^/mm^3^)	203.00 (147.00–276.00)	205.00 (148.00–273.50)	182.00 (83.50–307.25)	0.572
** Glucose (mmol/L)**	5.59 (4.83–6.98)	5.51 (4.81–6.78)	7.03 (4.98–12.22)	0.040
** Albumin (g/L)**	36 (31.15–39.68)	36.00 (32.00–40.00)	28.60 (23.68–34.88)	<0.001
** Blood urea nitrogen (mmol/L)**	4.6 0 (3.60–6.10)	4.55 (3.50–6.00)	5.60 (4.82–10.68)	0.002
** CRP (mg/L)**	37.10 (8.99–110.90)	36.15 (8.44–107.50)	130.50 (81.63–186.00)	0.001
** PCT**	0.10 (0.04–0.45)	0.08 (0.04–0.40)	1.29 (0.22–3.68)	<0.001
**LAMP**				
** Bacteria detection**	144 (39.34%)	136 (39.31%)	8 (40.00%)	0.951
** Virus detection**	153 (41.80%)	141 (40.75%)	12 (60.00%)	0.090
** Fungus detection**	71 (20.52%)	7 (35.00%)	78 (21.31%)	0.209
**PSI, n (%)**	77 (58–94)	75 (57–91)	97 (85–130)	<0.001
**CURB-65**	1 (0–1)	1 (0–1)	2 (1–2)	<0.001
**Severe CAP**	56 (15.30%)	37 (10.69%)	19 (95.00%)	<0.001

Data are presented as means ± standard error from the mean, or median (interquartile range) or n (%). COPD, chronic obstructive pulmonary disease; ARDS, Acute Respiratory Distress Syndrome; CRP, C-reactive protein; PCT, procalcitonin; LAMP, loop-mediated isothermal amplification; PSI, Pneumonia Severity Index; CURB-65 confusion, urea >7 mmol/L, respiratory rate ≥30 breaths/min, low blood pressure, and age ≥65 years.

### Statistical analysis for clinical factors with 30-day survival

Independent indicators were respectively introduced into univariate Cox proportional hazard regression analyses to investigate their associations with 30-day survival. The results showed that RR, HR, WBC, ALB, BUN, CRP, the PSI, and CURB-65 were associated with significantly high risk ratios, while no significant associations were found between 30-day survival and sex, age, mean arterial pressure, body temperature, hemoglobin levels, platelet counts, glucose, or PCT. When the significant independent variables were integrated into a multivariate Cox proportional hazards regression analysis, only RR [hazard ratio (95% CI): 1.150 (1.091–1.213)] and ALB [hazard ratio (95% CI): 0.907 (0.847–‍0.970)] were found to be strong independent predictors of 30-day survival (P<0.001 and P = 0.005, respectively). The hazard ratio of RR indicated that the risk of death was correlated with an increase in RR values, while ALB was the opposite—ALB was a protective factor, and the risk of mortality was negatively correlated with an increase in ALB levels. The results are summarized in Table 2.

**Table 2:** Cox regression analysis of risk factors associated with mortality.

	Univariate analysis		Multivariate analysis	
	Hazard ratio (95% CI)	P value	Hazard ratio (95% CI)	P value
**Male sex**	1.445 (0.555–3.760)	0.451		
**Age**	1.029 (0.999–1.061)	0.057		
**Mean arterial pressure**	0.989 (0.955–1.025)	0.554		
**Respiratory rate**	1.156 (1.108–1.207)	<0.001	1.150 (1.091–1.213)	<0.001
**Heart rate**	1.026 (1.004–1.048)	0.022		
**Body temperature**	1.001 (0.707–1.417)	0.997		
**White blood cell count**	1.062(1.009–1.117)	0.022		
**Hemoglobin level**	1.004 (0.987–1.022)	0.656		
**Platelet count**	0.999 (0.995–1.004)	0.774		
**Glucose**	1.098 (0.974–1.237)	0.126		
**Albumin**	0.874 (0.817–0.934)	<0.001	0.907 (0.847–0.970)	0.005
**Blood urea nitrogen**	1.123 (1.053–1.198)	<0.001		
**CRP**	1.006 (1.003–1.009)	<0.001		
**PCT**	1.009 (0.951–1.070)	0.768		
**PSI**	1.032 (1.019–1.045)	<0.001		
**CURB-65**	2.809 (1.841–4.287)	<0.001		
**Liver disease**	21.920 (0.005–101721.914)	0.474		
**Chronic renal disease**	21.339 (0.001–487402.490)	0.550		
**Malignant disease**	1.735 (0.232–12.958)	0.591		

CI, Confidence interval; CRP, C-reactive protein; PCT, procalcitonin; PSI, Pneumonia Severity Index; CURB-65, confusion, urea >7 mmol/L, respiratory rate ≥30 breaths/min, low blood pressure and age ≥65 years.

### Correlation between levels of ALB and RR, PSI, and CURB-65

**Fig 1:**
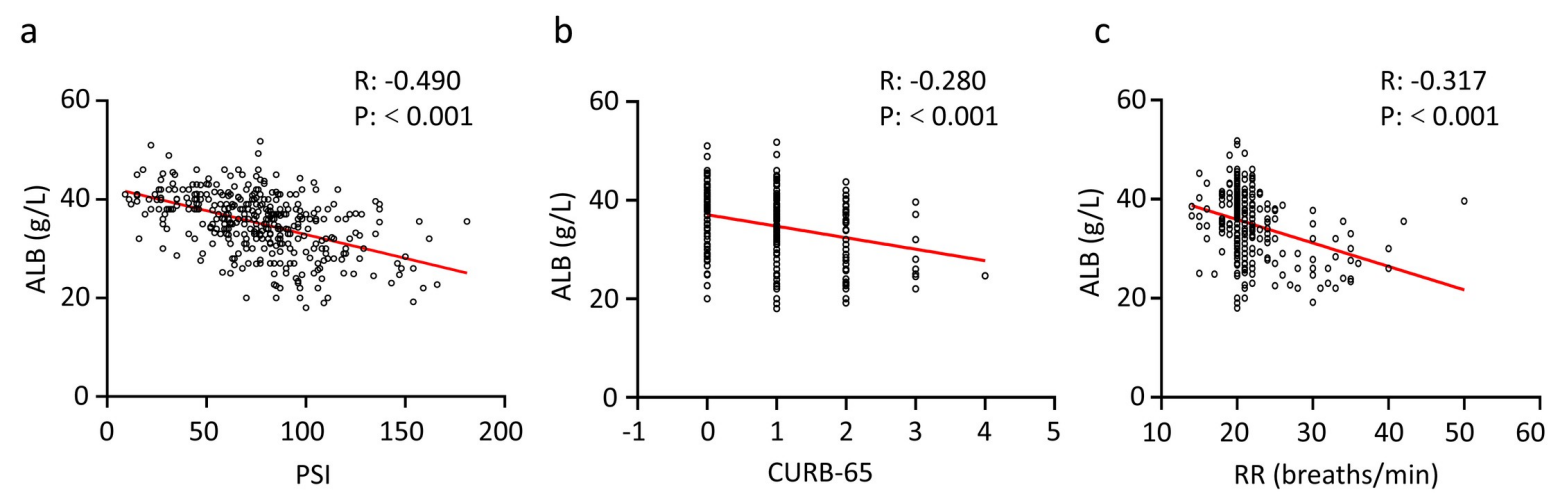
Correlation of serum ALB levels with two scoring systems and RR across 366 patients with CAP. R is the correlation coefficient. Fig 1A, 1B and 1C: Levels of ALB were negatively correlated with PSI, CURB-65, and RR. ALB, serum albumin; RR, respiratory rate.

The PSI and CURB-65 scoring systems were used to evaluate the severity of CAP. Respiratory rate and ALB showed associations with 30-day survival, as presented in Table 2. We further evaluated the correlation between ALB levels and RR, PSI, and CURB-65 scores. Because RR is an indicator in these two scoring systems, we did not investigate correlations of RR with the PSI and CURB-65 scores. As shown in Fig 1, ALB levels were moderately negatively correlated with the PSI score (R = -0.490, P<0.001), while it was mildly negatively correlated with CURB-65 scores (R = -0.280, P<0.001) and RR (R = -0.317, P<0.001). This data further confirmed that an increase in serum ALB was a protective factor.

### The prognostic value of ALB and RR in patients with CAP

**Fig 2:**
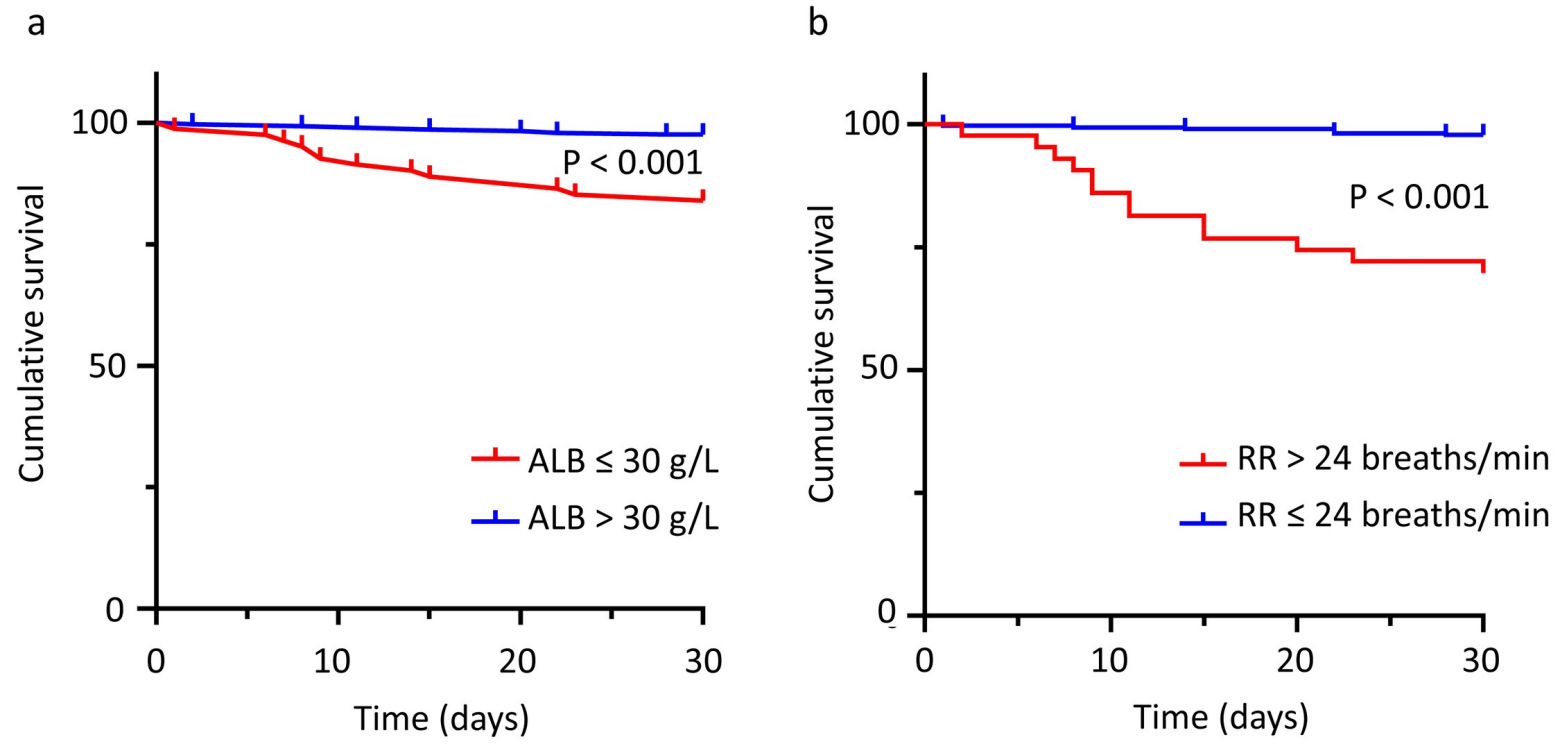
Kaplan–Meier analysis of 30-day mortality in patients with CAP. Analysis was stratified by serum ALB (A) and RR (B) levels. ALB, serum albumin; RR, respiratory rate.

Receiver operating characteristic curves of the RR, ALB, PSI, and CURB-65 scores were generated and produced independently (Table 3). The curves show that the threshold value of RR was 24 breaths/min for the prediction of mortality, with a sensitivity and specificity of 65.00% and 91.33%, respectively. The threshold values of ALB, PSI, and CURB-65 were 30 g/L, 83, and 1, respectively. Detailed results can be seen in Table 3. Overall, 20 (5.46%) patients with CAP died within 30 days. Kaplan–Meier curves were used to assess the relationship between RR and ALB levels in the prediction of 30-day mortality in patients with CAP (Fig 2). Respiratory rate and ALB were divided into two groups according to the thresholds from the ROC curves: RR (>24 breaths/min vs ≤24 breaths/min) and ALB (>30 g/L vs ≤30 g/L). The risk of death in groups with a RR >24 breaths/min and ALB ≤30 g/L was significantly higher than the risk of death in groups with a RR ≤24 breaths/min and ALB >30 g/L (P<0.001).

**Table 3:** AUC and thresholds for predicting non-survivors with survivors.

	Threshold	Sensitivity (%)	Specificity (%)	AUC	P value	95% CI Lower limit	Higher limit
**Respiratory rate(RR)**	>24 breaths/min	65.00	91.33	0.763	<0.001	0.629	0.897
**Albumin (ALB)**	≤30 g/L	65.00	80.35	0.762	<0.001	0.646	0.877
**PSI**	>83	85.00	63.87	0.790	<0.001	0.710	0.871
**CURB-65**	>1	60.00	85.55	0.784	<0.001	0.686	0.882

AUC, area under the curve; CI, confidence interval; PSI, Pneumonia Severity Index; CURB-65, confusion, urea > 7 mmol/L, respiratory rate ≥ 30 breaths/min, low blood pressure and age ≥ 65 years.

To further explore the predictive value of ALB for CAP prognosis in hospitalized patients, the patients with CAP were first divided into two groups according to the thresholds of the PSI, CURB-65, and RR. Each group was then divided into two sub-groups according to the threshold of ALB levels (Table 3). We evaluated the prognostic value of ALB levels at the time of admission to hospital for the different groups (Table 4). Patients with ALB levels ≤30 g/L had a significantly higher risk of 30-day mortality in the groups with a PSI score >83, CURB-65 ≤1, and RR ≤24 breaths/min (P = 0.012, P<0.001, P = 0.001, respectively). No significant difference was found in the groups with a PSI score <83, CURB-65 >1, and RR >24 breaths/min.

**Table 4:** 30-day mortality according to PSI, CURB-65 risk groups, RR and ALB in patients with CAP.

	PSI≤83 (n = 224)			PSI>83 (n = 142)		
	ALB≤30g/L (n = 21)	ALB>30g/L (n = 203)	P value	ALB≤30g/L (n = 60)	ALB>30g/L (n = 82)	P value
**30-day mortality**	1(4.8)	2(1.0)	0.257	12(20)	5(6.1)	0.012
	**CURB-65 ≤ 1 (n = 304)**			**CURB-65 > 1 (n = 62)**		
	ALB≤30g/L (n = 56)	ALB>30g/L (n = 248)	P value	ALB≤30g/L (n = 25)	ALB>30g/L (n = 37)	P value
**30-day mortality**	6(10.7)	2(0.8)	<0.001	7(28.0)	5(13.5)	0.276
	**RR≤24 breaths / min (n = 323)**			**RR>24 breaths / min (n = 43)**		
	ALB≤30g/L (n = 53)	ALB>30g/L n = (270)	P value	ALB≤30g/L (n = 28)	ALB>30g/L (n = 15)	P value
**30-day mortality**	5(9.4)	2(0.7)	0.001	8(28.6)	5(33.3)	1.000

CAP, community-acquired pneumonia; PSI, Pneumonia Severity Index; CURB-65, confusion, urea > 7 mmol/L, respiratory rate ≥ 30 breaths/min, low blood pressure and age ≥ 65 years; ALB, serum albumin; RR, respiratory rate.

**Fig 3:**
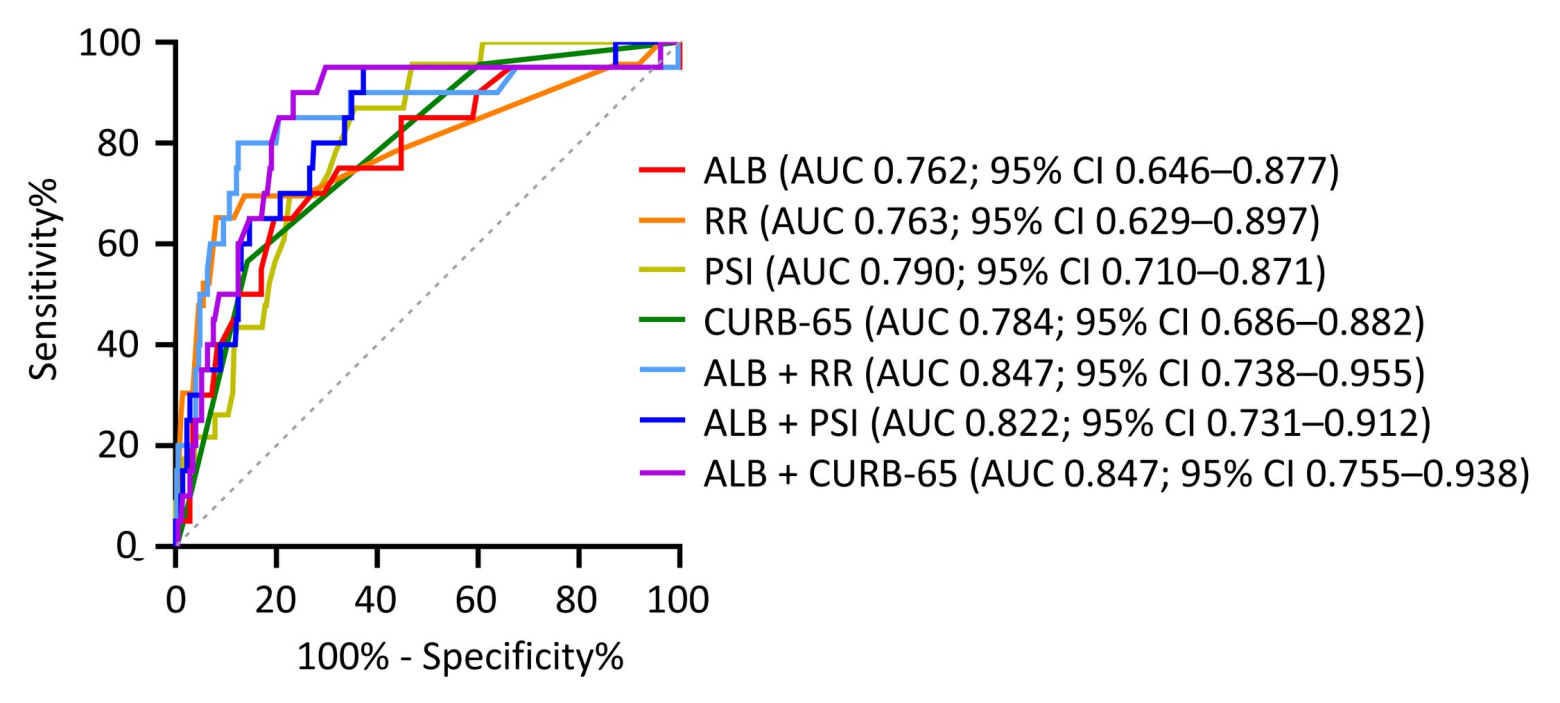
Receiver operating characteristic curve (ROC) analysis of various parameters in predicting CAP prognosis. AUC, area-under-the-curve; ALB, serum albumin; RR, respiratory rate; PSI, Pneumonia Severity Index Score; CURB-65, confusion, urea >7 mmol/L, respiratory rate ≥30 breaths/min, low blood pressure and age ≥65 years.

The ROC curves of the different indicators were combined to predict mortality. For the single indicator, the AUCs of the PSI, CURB-65, ALB, and RR were 0.790 (95% CI 0.710–0.871), 0.784 (95% CI 0.686–0.882), 0.762 (95% CI 0.646–0.877), and 0.763 (95% CI 0.629–0.897) for predicting mortality, respectively. The capacity of the combined indicators to predict 30-day mortality was higher than that of single indicators. The AUC for 30-day mortality for ALB levels combined with PSI scores was 0.822 (95% CI 0.731–0.912), while an AUC of 0.847 (95% CI 0.755–0.938) was calculated for ALB combined with CURB-65 scores. Furthermore, the AUC of ALB levels combined with RR was 0.847 (95% CI 0.738–0.955; Fig 3).

## Discussion

In this study, it was discovered that an increased RR and decreased ALB level were risk factors for mortality in patients with CAP, calculated by Cox regression analysis. The combined use of ALB levels with RR, the PSI, or CURB-65 significantly improved the accuracy of mortality prediction using ROC curves. Several conclusions and observations can be drawn from the results of this study, some of which are consistent with previously reported results, whereas other observations are novel.

First, within 24 hours of hospital admission, the two independent indicators of RR and ALB levels were screened and associated with 30-day mortality in patients with CAP by means of Cox multivariate regression analysis. Results were not entirely consistent with those reported previously [25–29]. Lee at al. used logistic regression analysis to screen and relate CRP and ALB to 30-day mortality [25]. Guo et al. identified that CRP and PCT were effective prognostic elements for use in hospitalized patients with CAP [6]. Other studies have described conventional indicators, such as lactate, to be effective elements for predicting mortality [17]. Two reasons for these differences among the various CAP studies exist. The indicators included in this study were different to those used in other studies. Additionally, the statistical models and methods used were also different. We used the Cox regression model rather than logistic regression.

Second, ALB levels had a mild to moderate negative correlation with RR, CURB-65, and the PSI. An increased ALB level demonstrated a protective effect. Therefore, it was possible to further improve the accuracy of predicting outcomes in patients with CAP by introducing ALB into these two scoring systems, or by combining ALB levels with RR.

Third, the Kaplan–Meier curves showed that a RR >24 breaths/min or ALB ≤30 g/L were associated with a significantly higher mortality risk. This finding regarding the relationship between serum ALB concentration and mortality in patients with CAP was similar to those from other studies [25,26,30]. Although the threshold values of ALB for the groupings were different, these papers all showed that the groups of patients with CAP that had a lower ALB level had a higher mortality risk.

Fourth, we further stratified patients according to the threshold values of CURB-65, the PSI, and RR, and found that only in groups with a PSI score >83, CURB-65 ≤1, or RR ≤24 breaths/min, was a decrease in ALB meaningful. This finding is different from that of research conducted by Viasus et al. [26], where patients with hypoalbuminemia had a significantly higher risk of 30-day mortality in both the low-risk and high-risk PSI and CURB-65 groups. The difference in the findings between the aforementioned and this study can be attributed to the difference in the number and severity of the admitted patients with CAP. In this study, the total number of patients was relatively small, the condition of most patients relatively mild, and 30-day mortality low.

Lastly, the combination of ALB levels with the PSI, CURB-65, or RR values significantly improved the accuracy of mortality prediction using ROC curves. This was the first time that the combined use of ALB and RR was studied, and it showed a significant improvement in predicting CAP prognosis compared to a single indicator. The combination of ALB with PSI and CURB-65 scores also significantly improved the predictive AUC-ROC of the PSI and CURB-65, which was consistent with the trends of previous research; however, the present study showed a more distinct improvement [25,26]. In this study, the AUC of the PSI and CURB-65 were found to be 0.790 and 0.784, respectively, and the AUC of ALB combined with the PSI and CURB-65 were 0.822 and 0.847, respectively, while in the study by Viasus et al., the AUC of the PSI and CURB-65 were 0.78 and 0.75, respectively, and the AUC of ALB combined with the PSI and CURB-65 was 0.8 and 0.78, respectively. Additionally, the AUC of the PSI was 0.76, and the AUC of ALB combined with the PSI was 0.79 in the study by Lee et al. Furthermore, the AUC-ROC suggested that the inclusion of ALB in the CURB-65 score was more significant than inclusion in the PSI score. A low serum ALB was identified as an independent prognostic variable by Lim et al., who originally proposed the CURB-65 scoring system, although ALB was not included in the final CURB-65 model due to it not being routinely tested in many hospitals at the time [12]. However, presently, serum ALB values are easy to detect at different levels of hospitals, and can be included in CURB-65 scores since they can help improve the prognosis of CAP. Another paper also showed that the expanded CURB-65 scoring system (including ALB <35 g/L) was more accurate for the evaluation of CAP severity than CURB-65 [31]. The AUC of ALB combined with RR was 0.847, which was equal to the AUC of the combination of ALB and CURB-65, and was superior to the AUC of the combination of ALB and the PSI. We recalculated the CURB-65 scores based on the adjusted RR (RR >24 breaths/min), termed CURB-65_RR>24. The ROC analysis showed that CURB-65_RR>24 had a better prognostic value for CAP than CURB-65 in our study (S1 Table). The application of ALB combined with RR in the prediction of the severity of CAP needs further verification.

There were several limitations to our study. First, 401 patients were diagnosed with CAP and admitted to various hospitals. Among these cases, 35 were excluded due to missing data, totaling 366 patients enrolled in this study. Data analysis may have had slight deviations because of missing data. Second, we admitted a total of 366 patients with less severe pneumonia. There were 304 cases with CURB-65 ≤1, accounting for 83% of the total enrolment, which was higher than in other studies [4,31,32]. The total number of deaths was 20, with a 30-day mortality of 5.46%, which was lower than that of previous studies of hospitalized patients with CAP [3–6,33]. This was likely due to a lower threshold for admission of patients with CAP, which may be derived from policies and regulations of the health care system. Third, we tried to build an early prognostic model, so the variables included in the Cox regression analysis were collected within 24 hours of admission. Since medical treatment was continuous and constantly adjusted based on changes in the disease status, it was not included in the analysis, although it was an important variable affecting prognosis.

In conclusion, this large, multi-center study focused on the predictive value of ALB and RR for the prognosis of patients with CAP within 24 hours of hospital admission. Serum ALB levels and RR exhibited a reliable prognostic value for CAP. The addition of serum ALB to the PSI and CURB-65 scoring could improve their prognostic accuracy in hospitalized patients with CAP. The combination of serum ALB and RR showed equal predictive value to the combination of serum ALB and CURB-65 for CAP prognosis in hospitalized patients.

## Supporting information

S1 Table ROC analysis of two parameters in predicting CAP prognosis. (DOCX)

## References

[1] Murray CJ, Lopez AD. Measuring the global burden of disease. The New England journal of medicine. 2013;369:448–57. 10.1056/NEJMra1201534 23902484

[2] Musher DM, Thorner AR. Community-acquired pneumonia. The New England journal of medicine. 2014;371:1619–28. 10.1056/NEJMra1312885 25337751

[3] Prina E, Ranzani OT, Torres A. Community-acquired pneumonia. Lancet. 2015;386:1097–108. 10.1016/S0140-6736(15)60733-4 26277247 PMC7173092

[4] Fang WF, Yang KY, Wu CL, Yu CJ, Chen CW, Tu CY, et al. Application and comparison of scoring indices to predict outcomes in patients with healthcare-associated pneumonia. Critical care. 2011;15:R32. 10.1186/cc9979 21247444 PMC3222068

[5] Song JH, Oh WS, Kang CI, Chung DR, Peck KR, Ko KS, et al. Epidemiology and clinical outcomes of community-acquired pneumonia in adult patients in Asian countries: a prospective study by the Asian network for surveillance of resistant pathogens. Int J Antimicrob Agents. 2008;31:107–14. 10.1016/j.ijantimicag.2007.09.014 18162378 PMC7134693

[6] Guo S, Mao X, Liang M. The moderate predictive value of serial serum CRP and PCT levels for the prognosis of hospitalized community-acquired pneumonia. Respiratory research. 2018;19:193. 10.1186/s12931-018-0877-x 30285748 PMC6167901

[7] Chalmers JD. Identifying severe community-acquired pneumonia: moving beyond mortality. Thorax. 2015;70:515–6. 10.1136/thoraxjnl-2015-207090 25877217

[8] Lim WS, Baudouin SV, George RC, Hill AT, Jamieson C, Le Jeune I, et al. BTS guidelines for the management of community acquired pneumonia in adults: update 2009. Thorax. 2009;64:iii1–55. 10.1136/thx.2009.121434 19783532

[9] Capelastegui A, Espana PP, Quintana JM, Areitio I, Gorordo I, Egurrola M, et al. Validation of a predictive rule for the management of community-acquired pneumonia. The European respiratory journal. 2006;27:151–7. 10.1183/09031936.06.00062505 16387948

[10] Fine MJ, Auble TE, Yealy DM, Hanusa BH, Weissfeld LA, Singer DE, et al. A prediction rule to identify low-risk patients with community-acquired pneumonia. The New England journal of medicine. 1997;336:243–50. 10.1056/NEJM199701233360402 8995086

[11] Neill AM, Martin IR, Weir R, Anderson R, Chereshsky A, Epton MJ, et al. Community acquired pneumonia: aetiology and usefulness of severity criteria on admission. Thorax. 1996;51:1010–6. 10.1136/thx.51.10.1010 8977602 PMC472650

[12] Lim WS, van der Eerden MM, Laing R, Boersma WG, Karalus N, Town GI, et al. Defining community acquired pneumonia severity on presentation to hospital: an international derivation and validation study. Thorax. 2003;58:377–82. 10.1136/thorax.58.5.377 12728155 PMC1746657

[13] Sungurlu S, Balk RA. The Role of Biomarkers in the Diagnosis and Management of Pneumonia. Clinics in chest medicine. 2018;39:691–701. 10.1016/j.ccm.2018.07.004 30390742

[14] Viasus D, Del Rio-Pertuz G, Simonetti AF, Garcia-Vidal C, Acosta-Reyes J, Garavito A, et al. Biomarkers for predicting short-term mortality in community-acquired pneumonia: A systematic review and meta-analysis. The Journal of infection. 2016;72:273–82. 10.1016/j.jinf.2016.01.002 26777314

[15] Menendez R, Mendez R, Aldas I, Reyes S, Gonzalez-Jimenez P, Espana PP, et al. Community-Acquired Pneumonia Patients at Risk for Early and Long-term Cardiovascular Events Are Identified by Cardiac Biomarkers. Chest. 2019;156:1080–91. 10.1016/j.chest.2019.06.040 31381883

[16] Charles PG, Wolfe R, Whitby M, Fine MJ, Fuller AJ, Stirling R, et al. SMART-COP: a tool for predicting the need for intensive respiratory or vasopressor support in community-acquired pneumonia. Clinical infectious diseases. 2008;47:375–84. 10.1086/589754 18558884

[17] Jo S, Jeong T, Lee JB, Jin Y, Yoon J, Park B. Validation of modified early warning score using serum lactate level in community-acquired pneumonia patients. The National Early Warning Score-Lactate score. The American journal of emergency medicine. 2016;34:536–41. 10.1016/j.ajem.2015.12.067 26803715

[18] Lepper PM, Ott S, Nuesch E, von Eynatten M, Schumann C, Pletz MW, et al. Serum glucose levels for predicting death in patients admitted to hospital for community acquired pneumonia: prospective cohort study. BMJ. 2012;344:e3397. 10.1136/bmj.e3397 22645184 PMC3362658

[19] Nowak A, Breidthardt T, Christ-Crain M, Bingisser R, Meune C, Tanglay Y, et al. Direct comparison of three natriuretic peptides for prediction of short- and long-term mortality in patients with community-acquired pneumonia. Chest. 2012;141:974–82. 10.1378/chest.11-0824 22135381

[20] Luo Q, Ning P, Zheng Y, Shang Y, Zhou B, Gao Z. Serum suPAR and syndecan-4 levels predict severity of community-acquired pneumonia: a prospective, multi-centre study. Critical care. 2018;22:15. 10.1186/s13054-018-1943-y 29368632 PMC5784729

[21] Gibot S, Cravoisy A, Levy B, Bene MC, Faure G, Bollaert PE. Soluble triggering receptor expressed on myeloid cells and the diagnosis of pneumonia. The New England journal of medicine. 2004;350:451–8. 10.1056/NEJMoa031544 14749453

[22] Ito A, Ishida T, Tachibana H, Ito Y, Takaiwa T. Serial procalcitonin levels for predicting prognosis in community-acquired pneumonia. Respirology. 2016;21:1459–64. 10.1111/resp.12846 27398948

[23] Liu D, Xie L, Zhao H, Liu X, Cao J. Prognostic value of mid-regional pro-adrenomedullin (MR-proADM) in patients with community-acquired pneumonia: a systematic review and meta-analysis. BMC infectious diseases. 2016;16:232. 10.1186/s12879-016-1566-3 27230573 PMC4881068

[24] Niederman MS, Mandell LA, Anzueto A, Bass JB, Broughton WA, Campbell GD, et al. Guidelines for the management of adults with community-acquired pneumonia. Diagnosis, assessment of severity, antimicrobial therapy, and prevention. American journal of respiratory and critical care medicine. 2001;163:1730–54. 10.1164/ajrccm.163.7.at1010 11401897

[25] Lee JH, Kim J, Kim K, Jo YH, Rhee J, Kim TY, et al. Albumin and C-reactive protein have prognostic significance in patients with community-acquired pneumonia. Journal of critical care. 2011;26:287–94. 10.1016/j.jcrc.2010.10.007 21129911

[26] Viasus D, Garcia-Vidal C, Simonetti A, Manresa F, Dorca J, Gudiol F, et al. Prognostic value of serum albumin levels in hospitalized adults with community-acquired pneumonia. The Journal of infection. 2013;66:415–423. 10.1016/j.jinf.2012.12.007 23286966

[27] Ito A, Ishida T, Tokumasu H, Washio Y, Yamazaki A, Ito Y, et al. Prognostic factors in hospitalized community-acquired pneumonia: a retrospective study of a prospective observational cohort. BMC pulmonary medicine. 2017;17:78. 10.1186/s12890-017-0424-4 28464807 PMC5414343

[28] Ma HM, Tang WH, Woo J. Predictors of in-hospital mortality of older patients admitted for community-acquired pneumonia. Age and ageing. 2011;40:736–41. 10.1093/ageing/afr087 21771744

[29] Artero A, Zaragoza R, Camarena JJ, Sancho S, Gonzalez R, Nogueira JM. Prognostic factors of mortality in patients with community-acquired bloodstream infection with severe sepsis and septic shock. Journal of critical care. 2010;25:276–81. 10.1016/j.jcrc.2009.12.004 20149587

[30] Miyazaki H, Nagata N, Akagi T, Takeda S, Harada T, Ushijima S, et al. Comprehensive analysis of prognostic factors in hospitalized patients with pneumonia occurring outside hospital: Serum albumin is not less important than pneumonia severity assessment scale. Journal of infection and chemotherapy. 2018;24:602–9. 10.1016/j.jiac.2018.03.006 29628384

[31] Liu JL, Xu F, Zhou H, Wu XJ, Shi LX, Lu RQ, et al. Expanded CURB-65: a new score system predicts severity of community-acquired pneumonia with superior efficiency. Scientific reports. 2016;6:22911. 10.1038/srep22911 26987602 PMC4796818

[32] Ilg A, Moskowitz A, Konanki V, Patel PV, Chase M, Grossestreuer AV, et al. Performance of the CURB-65 Score in Predicting Critical Care Interventions in Patients Admitted With Community-Acquired Pneumonia. Annals of emergency medicine. 2019;74:60–8. 10.1016/j.annemergmed.2018.06.017 30078659 PMC6359992

[33] Peyrani P, Arnold FW, Bordon J, Furmanek S, Luna CM, Cavallazzi R, et al. Incidence and Mortality of Adults Hospitalized With Community-Acquired Pneumonia According to Clinical Course. Chest. 2020;157:34–41. 10.1016/j.chest.2019.09.022 31610158

