# Supplementary material for: The prognostic value of serum albumin levels and respiratory rate for community-acquired pneumonia: A prospective, multi-center study
Source: PLoS One. 2021 Mar 4;16(3):e0248002. doi: 10.1371/journal.pone.0248002 (PMC7932099; doi:10.1371/journal.pone.0248002)
Supplement: S1 Table — (DOCX) [file pone.0248002.s001.docx]

S1 Table. ROC analysis of two parameters in predicting CAP prognosis

| Parameter | Threshold | Sensitivity (%) | Specificity (%) | AUC | P value | 95% CI |
| --- | --- | --- | --- | --- | --- | --- |
| CURB-65 | >1 | 60.00 | 85.55 | 0.784 | <0.001 | 0.686–0.882 |
| CURB-65_RR>24 | >1 | 70.00 | 84.39 | 0.813 | <0.001 | 0.769–0.851 |
